# Practical Application of Artificial Intelligence Technology in Glaucoma Diagnosis

**DOI:** 10.1155/2022/5212128

**Published:** 2022-07-31

**Authors:** Di Gong, Man Hu, Yue Yin, Tong Zhao, Tong Ding, Fan Meng, Yongli Xu, Yi Chen

**Affiliations:** ^1^Department of Ophthalmology, China-Japan Friendship Hospital, Beijing, China; ^2^Department of Ophthalmology, Beijing Children's Hospital, Capital Medical University, National Center for Children's Health, Beijing 100045, China; ^3^Beijing Institute of Ophthalmology, Beijing Tongren Hospital, Capital Medical University, Beijing Ophthalmology & Visual Science Key Lab, Beijing, China; ^4^Department of Mathematics, Beijing University of Chemical Technology, Beijing, China

## Abstract

**Purpose:**

By comparing the performance of different models between artificial intelligence (AI) and doctors, we aim to evaluate and identify the optimal model for future usage of AI.

**Methods:**

A total of 500 fundus images of glaucoma and 500 fundus images of normal eyes were collected and randomly divided into five groups, with each group corresponding to one round. The AI system provided diagnostic suggestions for each image. Four doctors provided diagnoses without the assistance of the AI in the first round and with the assistance of the AI in the second and third rounds. In the fourth round, doctor B and doctor D made diagnoses with the help of the AI and the other two doctors without the help of the AI. In the last round, doctor A and doctor B made diagnoses with the help of AI and the other two doctors without the help of the AI.

**Results:**

Doctor A, doctor B, and doctor D had a higher accuracy in the diagnosis of glaucoma with the assistance of AI in the second (*p*=0.036, *p*=0.003, and *p* ≤ 0.000) and the third round (*p*=0.021, *p* ≤ 0.000, and *p* ≤ 0.000) than in the first round. The accuracy of at least one doctor was higher than that of AI in the second and third rounds, in spite of no detectable significance (*p*=0.283, *p*=0.727, *p*=0.344, and *p*=0.508). The four doctors' overall accuracy (*p*=0.004 and *p* ≤ 0.000) and sensitivity (*p*=0.006 and *p* ≤ 0.000) as a whole were significantly improved in the second and third rounds.

**Conclusions:**

This “Doctor + AI” model can clarify the role of doctors and AI in medical responsibility and ensure the safety of patients, and importantly, this model shows great potential and application prospects.

## 1. Introduction

Glaucoma is a leading cause of irreversible blindness, and it afflicts 76 million people worldwide in 2020, with a prevalence rate of 3.5% [1]. It is widely recognized that early detection and treatment can preserve vision in afflicted individuals. However, glaucoma is asymptomatic in the early stages, as visual fields are not affected until 20–50% of corresponding retinal ganglion cells are lost [2, 3]. To this point, there is a need to enhance the capability to detect glaucoma and its progression and optimize treatment algorithms to preserve vision in patients with glaucoma.

Assessment of optic nerve head (ONH) integrity is the foundation for detecting glaucomatous damage. The ONH is a site where 1 million retinal ganglion cell axons converge on a space with an average area of 2.1–3.0 mm^2^ before radiating to higher visual pathways [4]. Given the variance in ONH anatomy [5], it is challenging to classify the glaucomatous disc in both clinical and screening settings. As the diagnosis of glaucoma is heavily hinged upon images, the artificial intelligence (AI) technique is expected to address some of these challenges.

Satisfactory prediction accuracy has been obtained in AI-assisted diagnosis of glaucoma based on fundus images [6–11]. Large training samples are needed since most existing deep learning (DL) systems only mine statistical information but not expert knowledge. In addition, the diagnostic logic of the DL systems in these studies is not transparent to physicians and the diagnostic results are often not interpretable. As a result, concerns about patient safety have arisen. Many controversies exist about the responsibility of medical behavior caused by AI technology [12]. AI systems face safety challenges in the forms of complex environments, unpredictable system behavior during periods of learning, and the uncertainty of human-machine interactions, all of which introduce significant variation in system performance. While these algorithms are powerful, they are often brittle because they may give inappropriate answers when fed with images outside their knowledge set [13]. Relative to most conventional information technology tools, AI applications may directly influence the diagnosis and management of a disease. Hence, this professional responsibility should be taken seriously and cautiously by doctors and computer scientists in this field. In radiology, Tang et al. [13] posited that AI applications should integrate and be interoperable with existing clinical workflows. AI applications should be clearly defined according to their role, type, and use cases in the clinical workflow. Therefore, it is not realistic for AI technology to completely replace the work of doctors. The combination of doctors and AI technology may be the way to solve the problem at present. There is evidence that AI can improve clinicians' performance as clinicians and AI working together outperformed either alone. For example, Lakhani and Sundaram have shown that a radiologist-augmented approach could enhance the performance of 2 deep neural networks by resolving their disagreements [14]. Many previous studies on AI in the field of ophthalmology targeted the reliability and success rate of AI technology [6,7,9,15–21], rather than the effect of cooperation between artificial intelligence technology and doctors in clinical work.

Previously, we established a DL system with a hierarchical structure (HDLS) based on a small number of samples [22]. This HDLS can comprehensively simulate the diagnostic thinking of human experts. In addition, this system was transparent and interpretable and the intermediate process of prediction can be visualized. On a small sample (200 cases of glaucoma and 200 cases of normal eyes), we verified that the AI algorithm could assist doctors in improving the accuracy of glaucoma diagnosis. In this study, we evaluated the learning curve of doctors in the process of diagnosing glaucoma using AI algorithms on a larger sample set (500 cases of glaucoma and 500 cases of normal eyes). In addition, we performed a systematic analysis of doctors' mistakes when using AI algorithms to assist in diagnosis, which will provide a more solid foundation for the clinical application of AI algorithms. The main purpose of this study is to explore the application forms and scenarios of artificial intelligence technology in glaucoma and the specific effect of cooperation between artificial intelligence and doctors.

## 2. Methods

### 2.1. Data Acquisition

The validation set of this study is a subset randomly selected from the extensive sample dataset of our previous study [22], including 500 cross-sectional (one image per patient at every time point) fundus images of glaucoma and 500 cross-sectional fundus images of normal eyes from Beijing Tongren Hospital. Eyes were diagnosed with glaucoma if there were both glaucomatous optic neuropathy (GON) and glaucomatous VF defects. GON was defined on optical coherence tomography (OCT) or stereoscopic color disc photography as either rim thinning or notching, peripapillary hemorrhages, or cup-disc ratio ≥0.6. Glaucomatous VF loss was diagnosed if any of the following findings were evident on two consecutive VF tests: a glaucoma hemifield test outside normal limits, pattern standard deviation (PSD) < 5%, or a cluster of three or more nonedged points in typical glaucomatous locations, all depressed on the pattern deviation plot at a level of *p* < 0.05, with one point in the cluster depressed at a level of *p* < 0.01 [23]. According to the diagnosis results of these fundus images by the AI system [22], the verification set contains 480 true positives, 20 false negatives, 460 true negatives, and 40 false positives. The entire validation set was randomly divided into five groups. Each group contained 96 true positives, 4 false negatives, 92 true negatives, and 8 false positives. These five groups of data were used as the validation set for the five stages in this study. This research followed the tenets of the Declaration of Helsinki.

All included subjects were adults (at least 18 years of age). Records being reviewed are diagnosis, medical history, and results from comprehensive ophthalmic examination, including visual acuity, intraocular pressure, slit-lamp biomicroscopy, gonioscopy, fundus, visual field, and optical coherence tomography (OCT) examination. In addition, fundus photographs (Nidek 3DX, Nidek, Japan) were collected. The inclusive criteria of fundus photography were as follows: (1) the photos were clear enough; (2) the scope of the images included the optic papilla and the nerve fibers around the optic papilla; (3) there was no occlusion on the photos that affected the diagnosis. The exclusion criteria of fundus photos were as follows: (1) the photos are not clear enough; (2) some factors such as fundus hemorrhage or cataracts lead to partial occlusion of the photos, so it is difficult to diagnose; (3) combined with other diseases of fundus such as age-related macular degeneration, diabetic retinopathy, ischemic optic papillopathy, pathological myopia or high myopia, and retinal venous occlusion. Ophthalmologists from Beijing China-Japan Friendship Hospital participated in the study. Two residents (doctor B and doctor D) with an average of 4.5 years of general ophthalmology work experience and 2 attending doctors (doctor A and doctor C) with an average of 11 years of general ophthalmology working experience participated in the study. The main purpose of setting up two groups of doctors (residents and attending doctors) is to avoid the deviation of research results caused by doctors' working years. They all learned glaucoma knowledge in the learning stage and diagnosed and treated glaucoma patients in the process of work. However, they have not been engaged in the diagnosis and treatment of glaucoma for a long time. The four doctors are not glaucoma specialists.

### 2.2. Data Processing of AI

The HDLS AI system can extract the anatomical characteristics of the fundus images, including the optic disc (OD), optic cup (OC), and the retinal nerve fiber layer defects (RNFLD), to realize automatic diagnosis of glaucoma. This AI system includes three modules: prediagnosis, image (OD, OC, and RNFLD) segmentation, and final diagnosis based on expert knowledge. Among them, the first two modules are designed based on deep learning [24]. In the final diagnosis module, the segmentation of OD and OC is used to extract features mean cup-to-disk ratio (MCDR) and ISNT (inferior, superior, nasal, and temporal) score. Then, a two-dimensional classification line is established by support vector machines (SVM) [25]. The final diagnostic criteria are as follows: (1) if there is an RNFLD, then it is predicted as glaucoma; (2) if there is no RNFLD, then make a prediction based on the two-dimensional classification line.

### 2.3. Experimental Procedures

In the first round of tests, the first group of fundus images was randomly scrambled. AI and four doctors gave diagnosis advice, respectively. In the second round of tests, the second group of fundus images was randomly scrambled. The diagnosis of AI was given to four doctors for reference, and then the diagnosis opinions of four doctors were obtained. The third round of the test was the same as the second round, in order to make doctors more familiar with AI. In the fourth round, the fourth group of fundus images was randomly scrambled. The diagnosis of AI was only given to residents, not attending doctors. In this way, the diagnosis of residents under the condition of AI assistance was obtained as well as the independent diagnosis opinions of attending doctors. In the final round, the fifth group of fundus images was randomly scrambled. The diagnosis of AI was given to two doctors (one resident and one attending doctor) who had a lower accuracy in the first three rounds of tests. An independent diagnosis was obtained from the other doctors. The process is shown in Table 1.

### 2.4. Statistical Analysis

In the five rounds of testing, the sensitivity, specificity, and accuracy of doctors and AI were obtained. In the analysis, we analyze not only the accuracy of individual doctors but also the overall accuracy of doctors as a whole. When the whole group of doctors was judged to be correct, the judgment of each doctor in the group was required to be right. Otherwise, it would be regarded as the error of the doctor group. This is a relatively strict criterion. In the second and third rounds, the number of cases with inconsistent AI diagnosis and doctor diagnosis was calculated, respectively. At the same time, the numbers of cases with AI consistent with doctors' diagnoses were also calculated. The ratio of the two indicated the doctors' dependency on AI. The chi-square test was used to compare each group's sensitivity, specificity, and accuracy. Data were analyzed using SPSS 26.0 software (SPSS, Los Angeles, CA, USA). A value of *p* < 0.05 was considered to be statistically significant.

## 3. Results

The sensitivity, specificity, and accuracy of AI in the diagnosis of glaucoma were 96%, 92%, and 94% in each round, respectively. The sensitivity, specificity, and accuracy of the four doctors are shown in Table 2.

**Figure 1:**
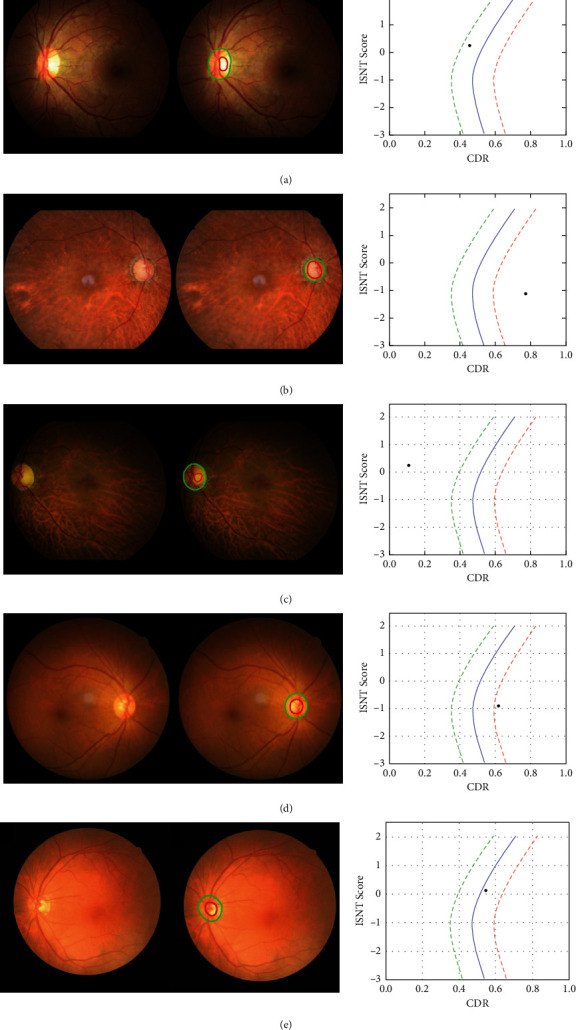
(a) A normal person: AI and four doctors all diagnosed correctly. (b) A patient with advanced glaucoma: AI and four doctors diagnosed correctly. (c) A normal person: AI diagnosis is correct, but the four doctors' diagnosis was wrong. The reason for the wrong diagnosis is the curved blood vessels under the optic disc, and AI can help doctors correct the diagnosis. (d) A normal person: the AI diagnosis is wrong. The four doctors are not affected by the AI diagnosis, and the diagnosis is correct. The reason for AI diagnosis error may be that the image resolution is slightly low, and the doctor can correct AI diagnosis in this instance. (e) An early glaucoma patient: both AI and a doctor correctly diagnosed. The other three doctors were all wrong. Then, AI can correct the doctor's diagnosis.

A group of fundus photographs of doctors and AI's diagnostic results are shown in Figure 1.

### 3.1. Accuracy of Individual Doctors

In the first round, there was no difference between the accuracy rate of doctor C and the accuracy rate of AI (*p*=0.727) and the accuracy rate of the other three doctors was not as good as AI (*p* ≤ 0.000, *p* ≤ 0.000, and *p* ≤ 0.000).

In the second round, although there was no significant difference between the accuracy of four doctors and AI (*p*=0.375, *p*=1.000, *p*=0.375, and *p*=0.581), the accuracy rate of diagnosis of four doctors was increased, of which two doctors were more accurate than AI. The correct rates of doctor A, doctor B, and doctor D were higher than those of the first round, and the difference was statistically significant (*p*=0.036, *p*=0.003, and *p* ≤ 0.000). Although the accuracy of doctor C was higher than that of the first round, the difference was not statistically significant (*p*=0.283).

In the third round, doctor B, doctor C, and doctor D had higher accuracy than AI and doctor A had the same accuracy as AI, but there was no significant difference (*p*=1.000, *p*=0.727, *p*=0.344, and *p*=0.508). The diagnostic accuracy of doctor D was the same as that of the second round, and the diagnostic accuracy of the other three doctors was improved compared to the second round. Still, there was no significant difference (*p*=0.456, *p*=0.467, and *p*=0.654).

There were still three doctors (doctor B, doctor C, and doctor D) with higher diagnostic accuracy than AI in the fourth round. After losing the assistance of AI, the correct rate of doctor A was lower than that of AI, while doctor C had higher accuracy than AI. However, there was still no significant difference between them and AI in accuracy (*p*=0.332, *p*=0.082, *p*=0.804, and *p*=0.334). The accuracy rates of doctor A and doctor C in the fourth round were higher than those in the first round, but there was no statistical difference (*p*=0.067).

In the final round, the diagnostic accuracy of two doctors (doctor B and doctor C) was higher than that of AI. The diagnostic accuracy of doctor A was the same as that of AI with the assistance of AI. Also, the diagnostic accuracy of doctor D was lower than that of AI without the assistance of AI. However, there was no significant difference between them and AI in accuracy (*p*=1.000, *p*=0.453, *p*=0.774, and *p*=0.754). The accuracy rate of doctor D in the fifth round was higher than that in the first round, and the difference was statistically significant (*p*=0.005). The accuracy rate of doctor A in the fifth round was higher than that in the first round, and the difference was not statistically significant (*p*=0.145).

**Figure 2:**
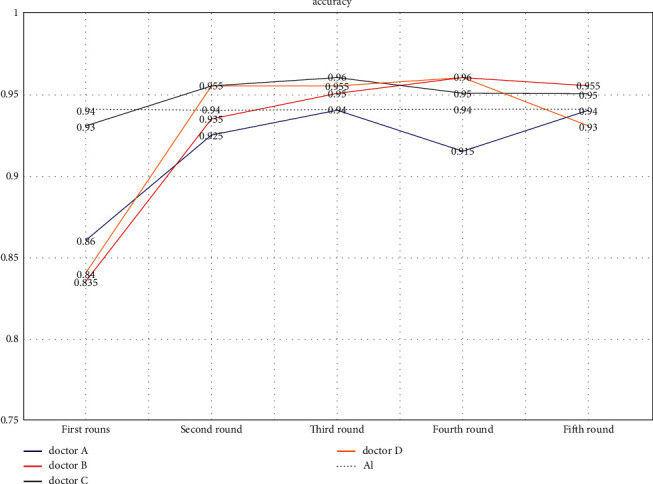
In the first round, the accuracy of 4 doctors was lower than that of AI. In the second round, with the help of AI, the accuracy of doctor C and doctor D was higher than that of AI, while the other 2 doctors had a lower accuracy with the help of AI. In the third round, with the help of AI, the accuracy of doctor A was the same as AI, while the accuracy of the other three doctors with the help of AI was higher than AI. In the fourth round, without the help of AI, the accuracy of doctor A was lower than AI, while the accuracy of the other three doctors (Doctor C did not have the help of AI, while doctor B and doctor D had the help of AI.) was higher than AI. In the fifth round, without the help of AI, the accuracy of doctor D was lower than AI. With the help of AI, the accuracy of doctor A was the same as AI, while the other two doctors (Doctor C did not have the help of AI, while doctor B had the help of AI.) had a higher accuracy.

**Figure 3:**
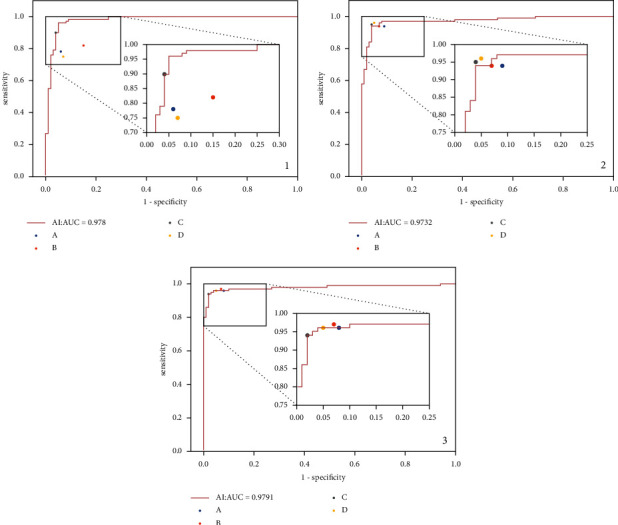
(a–c) The ROC curve of AI diagnosis and the sensitivity and specificity of four doctors' diagnosis in the first, second, and third rounds. It can be seen from (a) that, in the first round, the AUC of AI diagnosis was 0.978. Among the four doctors, the diagnostic accuracy of doctor C was roughly equal to that of AI and the diagnostic accuracy of doctors A, B, and D was significantly lower than that of AI. In the second stage, the AUC of AI diagnosis was 0.973. Among the four doctors, the diagnostic accuracy of doctor C and doctor D was slightly higher than that of AI, the diagnostic accuracy of doctor B was roughly the same as that of AI, and the diagnostic accuracy of doctor A was slightly lower than that of AI. In the third round, the AUC of AI diagnosis was 0.979. Among the four doctors, the diagnostic accuracy of doctor B was slightly higher than that of AI, and the diagnostic accuracy of doctors A, C, and D was roughly the same as that of AI.

From the line chart (Figure 2), it could be found that the accuracy of the four doctors improved after AI assistance. Except for doctor A, the accuracy rate of the other three doctors was higher than that of AI in the third round. From the ROC curve (Figure 3), the diagnostic accuracy of the three doctors of A, B, and D in the second and third rounds increased significantly compared with the first round. Moreover, in the second round, the diagnostic accuracy of doctor C and doctor D was higher than that of AI. Also, in the third round, the diagnostic accuracy of doctor B was higher than that of AI.

### 3.2. Accuracy Rate of Doctor Group

**Figure 4:**
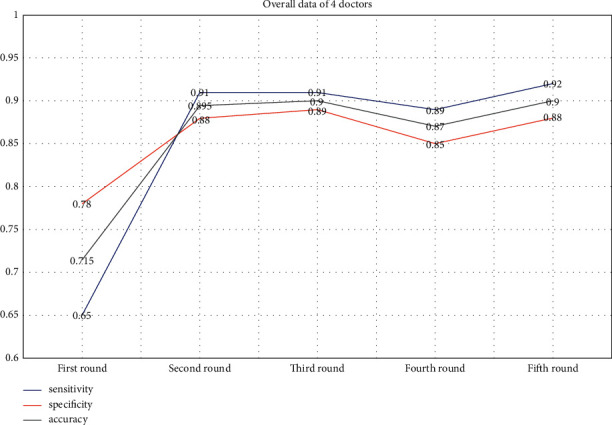
The overall sensitivity, specificity, and accuracy of doctors were greatly improved with the help of AI, and the change trend was consistent. Especially in the third round, the overall accuracy of doctors reached that of AI. Due to the strict requirements for doctors as a whole, this achievement was very commendable. In the fourth and fifth rounds, it could be seen that doctors with low accuracy combined with AI could significantly improve the overall diagnostic accuracy of doctors.

In the first round, the overall accuracy rate of 4 doctors was 71.5%. Compared with the accuracy of AI, it was lower with a statistically significant difference (*p* ≤ 0.000). In the second round, the overall correct rate of 4 doctors increased to 89.5%, but it is still not as good as that of AI. The difference was statistically significant (*p* ≤ 0.004). However, the overall correct rate of 4 doctors was significantly higher than that of the first round and the difference was statistically significant (*p* ≤ 0.000). In the third round, the overall accuracy rate of 4 doctors also improved, and it was 90%. Also, there was no significant difference with AI (*p*=0.057). In the fourth round, because the attending doctors did not have the assistance of AI, the overall correct rate of 4 doctors (87%) was not as good as that of AI and the difference was statistically significant (*p*=0.004). However, compared with the first round, the overall accuracy of 4 doctors was still improved and there was a significant statistical difference (*p* ≤ 0.000). In the final round, doctor C and doctor D did not have the assistance of AI, but the overall correct rate of 4 doctors was the same as that in the third round. Also, there is no statistically significant (*p*=0.057) between the overall correct rate of 4 doctors and that of AI in the final round. From the line chart (Figure 4), it could be found that with the assistance of AI, the overall diagnostic accuracy, sensitivity, and specificity of doctors were improved.

The overall accuracy rate of attending doctors (82.0%) was higher than that of residents (75.5%), but the difference was not statistically significant (*p*=0.092) in the first round. In the fourth round, with the assistance of AI, the accuracy rate of residents (95.0%) was higher than that of attending doctors (88.5%) and there was a significant statistical difference (*p*=0.004).

In the first three rounds of tests, compared with doctor A and doctor B, doctor C and doctor D had higher accuracy. In the fifth round, divide doctor A and doctor B into a group and doctor C and doctor D into the other group. Doctor A and doctor B had the assistance of AI, and doctor C and doctor D did not. The accuracy of doctor A and doctor B was 93%, and the accuracy of doctor C and doctor D was 91.5%. There was no significant difference in the accuracy between the two groups (*p*=0.508) in the final round. However, in the first round, the overall accuracy of doctor A and doctor B (77.5%) was lower than that of doctor C and doctor D (86%) and there was a significant difference (*p* ≤ 0.000).

### 3.3. Analysis of Doctor Dependency on AI

In the second round and the third round, the number of cases with inconsistent AI diagnosis and doctor diagnosis was calculated, respectively; cases of doctor A, doctor B, doctor C, and doctor D were 19 (4.75%), 13 (3.25%), 15 (3.75%), and 21 (5.25%), respectively. At the same time, the numbers of cases with AI consistent with doctors' diagnosis were also calculated. The ratio of the two indicated the doctors' dependency on AI. The four doctors' dependency on AI was 4.99%, 3.36%, 3.90%, and 5.54%, respectively. The lower the value, the more dependent the doctor was on AI's diagnosis. In the second round and the third round, the accuracy of the four doctors was ranked as follows: doctor C, doctor D, doctor B, and doctor A. There was no corresponding relationship between the accuracy and the dependency of the four doctors.

### 3.4. Sensitivity and Specificity

In the first round, the sensitivity of doctor A, doctor B, and doctor D was lower than that of AI (*p* ≤ 0.000, *p*=0.002, and *p* ≤ 0.000). There was no significant difference between the sensitivity of doctor C and AI (*p*=0.096). In the second and third round, the sensitivity of the four doctors was improved with the assistance of AI. For both the second and third rounds, there was no significant difference between the sensitivity of doctors with that of AI (*p*=0.516, *p*=0.516, *p*=0.773, and *p*=1.000). Although there was no significant difference, the sensitivity of doctor B is higher than that of AI in the second round. The overall sensitivity of doctors was lower than that of AI in the first round (*p* ≤ 0.000). In the second and third round, the overall sensitivity of doctors was improved. Whether it is the second round or the third round, there was no significant difference between the overall sensitivity of doctors and that of AI (*p*=0.152).

In the first round, the specificity of doctor A, doctor B, doctor C, and doctor D was not significantly different from that of AI (*p*=0.579, *p*=0.121, *p*=0.234, and *p*=0.788), but the overall specificity of doctors was lower than that of AI (*p*=0.006). In the second and third round, with the assistance of AI, the specificity of doctor B, doctor C, and doctor D increased, while the specificity of doctor A decreased slightly, but these changes were not statistically significant (*p*=0.459, *p*=0.331, *p*=0.314, and *p*=0.248). In the second and third rounds, the overall specificity of doctors was improved. Also, there was no significant difference between the overall specificity of 4 doctors with that of AI (*p*=0.346) in the second and third rounds. However, there was a significant increase from the overall specificity of 4 doctors in the first round to that in the second or third round (*p*=0.006 and *p* ≤ 0.000).

**Figure 5:**
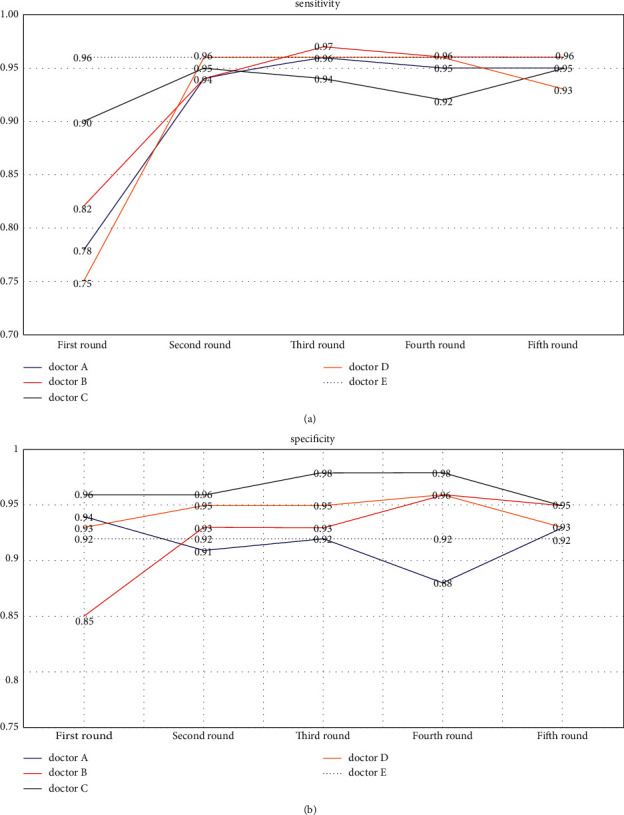
(a) The sensitivity of the four doctors in the first round was lower than that of AI. In the second and third round, the sensitivity of doctors was significantly improved. Among them, the sensitivity of doctor B exceeded that of AI in the third round. Without the assistance of AI, the sensitivity of doctor C decreased in the fourth round, and the same thing happened for doctor D in the fifth round. (b) In the first round, the specificity of three doctors was higher than that of AI and only doctor B's was lower than that of AI. In the second and third round, the specificity of doctors did not change much. Besides doctor A, the specificity of three doctors was higher than that of AI. In the fourth and fifth round, there was no significant trend change in specificity. The reason was that doctors had a great grasp of the diagnosis for the late glaucoma. Whether AI was involved or not, this phenomenon did not change much.

From the line chart (Figures 4 and 5), we can see the change of sensitivity and specificity of the four doctors individually and combined.

## 4. Discussion

For AI diagnosis of ophthalmic diseases based on fundus images, considerable progress was achieved in recent years. DL algorithms have been designed to diagnose diseases such as diabetic retinopathy and age-related macular degeneration based on fundus images [26–29]. In addition, the DL models have also been used to predict cardiovascular risk factors based on fundus images [30]. However, in the above studies, the DL algorithms were black-box models. Although these models can achieve high enough prediction accuracy, physicians could not interoperate the deduction process of the results.

Specifically for glaucoma detection, some deep learning methods have been proposed. Liu et al. used deep learning for glaucoma diagnosis, and a heat map showed that the features were extracted from the optical disc (OD) area. However, how the algorithm used these features to make a diagnosis was incomprehensible to physicians [8]. Fu et al. used the deep learning method to segment the optical disc (OD) and optical cup (OC) and used the vertical cup-to-disc ratio (VCDR) as an indicator to diagnose glaucoma [31]. However, underlying these exciting advances is the critical notion that these algorithms do not replace human doctors' breadth and contextual knowledge. Even the best algorithms would need to integrate into existing clinical workflows in order to improve patient care. In one study, algorithm-assisted pathologists demonstrated higher accuracy than either the algorithm or the pathologist alone [32]. In particular, algorithm assistance significantly increased the sensitivity of detection for micrometastases (91% vs. 83%, *p*=0.02) [18]. Another study found that radiologists combined with artificial intelligence technology could further improve the accuracy of diagnosis of *tuberculosis* on chest X-rays [8]. This showed that doctors combined with AI had a good application prospect. This can not only solve the responsibility problem of AI in medical behavior, but also the ethical problem. However, few similar studies are conducted in ophthalmology. At present, there are two AI studies on glaucoma, which compare the accuracy of AI with that of doctors, but do not apply AI technology in doctors' diagnosis [9, 18]. In our study, we not only analyze the sensitivity, specificity, and accuracy of AI technology compared with doctors but also compare the changes in doctors' diagnosis accuracy with or without the assistance of AI technology, so that we can analyze the value of AI technology in glaucoma diagnosis.

In the first round of the test, we found that only the accuracy of doctor C could reach the level of AI, while the other three doctors' accuracy was not as good as AI. However, in the second round, the diagnostic accuracy of the four doctors improved, which benefited from the assistance of AI. Especially for doctor A, doctor B, and doctor D, their benefits are more salient, and the diagnostic accuracy of each doctor had reached the level of AI. It showed that AI could make immediate and tangible improvement in individual doctor's accuracy. In the third round, the diagnostic accuracy rate of the four doctors also maintained a high level, which further proved the obvious and stable role of AI in helping doctors. The diagnostic accuracy of at least one doctor was higher than that of AI in the second or third round. The effect of “AI + Doctor” was greater than that of a doctor or AI, although statistical analysis showed no difference between the accuracy of “AI + Doctor” and AI. The reason was that the 94% diagnostic accuracy of AI was too high, so it was challenging to achieve a statistically higher diagnostic accuracy.

The independent diagnostic accuracy of doctor A and doctor C in the fourth round was higher than that in the first round. The independent diagnostic accuracy of doctor D in the fifth round was also higher than that in the first round. It showed that artificial intelligence not only had the function of assisting doctors but also had a good teaching function. In particular, for doctor D, the difference was statistically significant. The results showed that the improvement of doctor D was the most conspicuous. However, the progress for doctor A and doctor C did not have a statistically significant difference due to the high accuracy in the first round. Doctor B, who cannot be counted, has no independent diagnosis data in the later stage.

AI could also improve the overall accuracy of doctors. In the first round, the overall diagnostic accuracy of doctors was not as good as AI, only 71.5%. In the second round, the accuracy rate reached 89.5%. Although there was no significant statistical difference, the improvement in accuracy rate was substantial. In the third round, the overall accuracy rate of doctors increased again, reaching 90%. In the third round, the overall accuracy of doctors had reached the level of AI, which was a qualitative leap. In calculating the overall accuracy of doctors, we adopt a stringent standard (Only when the judgment of each doctor in the group was correct, the whole group of doctors was counted as correct. Otherwise, it would be regarded as the error of the doctor group.). It was conducive to obtaining more reliable research data for both patients and doctors. We believed that the standard would have a positive impact on the trust of both patients and doctors in AI.

In the fourth round, we designed a comparative test between residents and attending doctors. Due to the help of AI, the overall accuracy rate of residents was higher than that of attending doctors and the difference was statistically significant. However, in the first round, the overall accuracy rate of residents was lower than that of attending doctors, although the difference was not statistically significant. This shows that AI-assisted diagnosis is very meaningful and can rapidly improve the glaucoma diagnosis level of junior ophthalmologists. For example, in glaucoma screening, young ophthalmologists can refer to AI's diagnosis suggestions, which can improve the diagnostic accuracy for glaucoma, improve the screening efficiency, and avoid the waste of medical resources. In the fifth round, we designed a comparative test between doctors with high accuracy and those with low accuracy. We found that, with the help of AI, the overall diagnostic accuracy of doctors with low accuracy could reach the level of doctors with high accuracy. In the first round, the overall correct rate of the two doctors with low correct rate was lower than that of the two doctors with high correct rate and the difference was statistically significant. This showed that AI was more helpful to doctors with lower diagnostic accuracy. This could also be seen from the overall diagnostic accuracy of doctors in the fourth and fifth rounds. The overall accuracy of doctors in the fifth round was higher than that in the fourth round because the two doctors with low accuracy got the help of AI.

In the first round, we found that the diagnostic sensitivity of the four doctors was generally lower than the specificity and the gap was huge. In fact, this was very easy to understand. Ophthalmologists with certain clinical experience had greater confidence in the diagnosis of advanced glaucoma patients with typical signs. However, it was easy to miss the diagnosis of early glaucoma patients because many glaucoma patients in early stages had no signs [2,3]. In terms of clinical work, low sensitivity meant a high rate of missed diagnosis for glaucoma patients in the early stages. Therefore, it was imperative to improve the sensitivity of diagnosis. The improvement of sensitivity meant that doctors could make early diagnoses and treatments of glaucoma patients, so as to better protect the visual function of glaucoma patients. With the help of AI, we could see that the diagnostic sensitivity of the four doctors had improved prominently. Not only that, the overall sensitivity of doctors was significantly enhanced with the help of AI in the second round and third round. In the fourth round and fifth round, the overall diagnostic sensitivity of the four doctors was still high, even if there were two doctors without the help of AI. With the help of AI, the improvement of doctors' diagnostic sensitivity was prominent, which was good news for glaucoma patients. In addition, with improved diagnostic sensitivity, the false positive and false negative were reduced. Those advancements improved the efficiency of glaucoma diagnostic behavior and avoided the waste of medical resources.

The four doctors' dependency on AI diagnosis results was not the same, with doctor B's being the highest and that of doctor D the lowest. At the same time, doctor C had the highest accuracy and doctor A had the lowest accuracy. There was no corresponding relationship between the diagnostic accuracy of the four doctors and their acceptance of AI. This showed that although the recognition of AI diagnostic results of the four doctors was not completely consistent, the diagnostic accuracy of the four doctors had been coherently improved with the help of AI. The cooperation between doctors and AI has improved the diagnosis of glaucoma. We thought that it was of great significance to patients. Medical decisions need to be made responsibly, but machines are incapable of fulfilling humanistic and ethical tasks. Therefore, the “AI + Doctor” model allows two systems to complement each other. It improves the accuracy and sensitivity of diagnosis while including human thinking and judgment. Keane PA and Topol EJ pointed out that clinicians will have an increasingly important role in this AI revolution that will ultimately lead to countless benefits for our patients [33].

Despite the promising results, our study has the following limitations. First, the diagnostic evidence of AI system did not include other factors besides optical disc (OD), optical cup (OC), and retinal nerve fiber layer defect (RNFLD), such as hemorrhage and peripapillary atrophy. Although the prediagnostic module may incorporate these factors, it is not explicitly expressed in the final diagnosis result. In addition, in our deep learning model, every fundus image was resized to a resolution of 512 × 512 given the limitations in the graphic processing unit's (GPU) computational power. As a result, the fine texture details of the retinal fiber layer are partially lost in the compressed fundus images, which affected the accuracy of the segmentation module in detecting RNFLD. Our study also has a relatively small sample size and a small number of participating doctors. We would continue to enhance our data collation and collection in the follow-up works. Moreover, it was difficult to achieve the average distribution of early glaucoma, middle glaucoma, and late glaucoma. Although we excluded patients with pathological myopia, a certain proportion of myopia in some normal subjects and glaucoma patients would have a certain impact on the research results. In addition, in clinical practice, only fundus color photography can be used to determine whether patients have glaucoma. This scenario is rare. Therefore, the specific effect of this cooperation model applied to clinical work needs to be verified. However, this cooperation model can have good application value and prospect in glaucoma screening. Therefore, we believe that screening may be the first important application scenario of AI diagnostic technology in glaucoma diagnosis, and glaucoma screening is also very important. In addition, even without the participation of AI, doctors conduct five rounds of glaucoma diagnosis tests alone, which has a certain learning effect. This learning effect itself will lead to the improvement of the correct diagnosis rate of glaucoma. In the later research, we should further analyze the role of this self-learning effect.

Taken together, we believe that the combination of doctors and artificial intelligence technology can not only improve the accuracy of medical decision-making and the quality of medical services but also improve the reliability of artificial intelligence medical decision-making and partially solve moral disputes. At the same time, individual doctors learn and progress faster through the “doctor + artificial intelligence” mode. The dual mode accelerates and improves the efficiency of medical education process. This cooperation model will first play an important role in glaucoma screening.

## Figures and Tables

**Table 1:** Process diagram of test.

	First round	Second round	Third round	Fourth round	Fifth round
Doctor A (attending doctor)	Independent	AI assisted	AI assisted	Independent	AI assisted
Doctor B (resident)	Independent	AI assisted	AI assisted	AI assisted	AI assisted
Doctor C (attending doctor)	Independent	AI assisted	AI assisted	Independent	Independent
Doctor D (resident)	Independent	AI assisted	AI assisted	AI assisted	Independent

The diagnosis process of four doctors in the study. “Independent” means that doctors have no reference for AI results. “AI assisted” indicates that the doctor knows the diagnostic result of AI before making the diagnosis.

**Table 2:** Diagnosis data of four doctors.

		First round	Second round	Third round	Fourth round	Fifth round
Doctor A	Sensitivity	78%	94%	96%	95%	95%
	Specificity	94%	91%	92%	88%	93%
	Accuracy	86%	92.5%	94%	91.5%	94%

Doctor B	Sensitivity	82%	94%	97%	96%	96%
	Specificity	85%	93%	93%	96%	95%
	Accuracy	83.5%	93.5%	95%	96%	95.5%

Doctor C	Sensitivity	90%	95%	94%	92%	95%
	Specificity	96%	96%	98%	98%	95%
	Accuracy	93%	95.5%	96%	95%	95%

Doctor D	Sensitivity	75%	96%	96%	96%	93%
	Specificity	93%	95%	95%	96%	93%
	Accuracy	84%	95.5%	95.5%	96%	93%

Doctors	Sensitivity	65%	91%	91%	89%	92%
	Specificity	78%	88%	89%	85%	88%
	Accuracy	71.5%	89.5%	90%	87%	90%

## Data Availability

The article contains the basic data of this study.
